# Development of an athyroid mouse model using ^131^I ablation after preparation with a low-iodine diet

**DOI:** 10.1038/s41598-017-13772-8

**Published:** 2017-10-16

**Authors:** Ji Min Oh, Ho Won Lee, Senthilkumar Kalimuthu, Prakash Gangadaran, Se Hwan Baek, Man-Hoon Han, Chae Moon Hong, Shin Young Jeong, Sang-Woo Lee, Jaetae Lee, Byeong-Cheol Ahn

**Affiliations:** 1 Department of Nuclear Medicine, Kyungpook National University and Hospital, Daegu, South Korea; 2 Department of Pathology, Kyungpook National University and Hospital, Daegu, South Korea; 3 Daegu-Gyeongbuk Medical Innovation Foundation (DGMIF), 80 Chembok-ro, Dong-gu, Daegu, South Korea

## Abstract

We optimized the protocol for thyroid ablation in living mice using radioactive iodine (RAI) and a low-iodine diet (LID). To examine the effect of LID on thyroid ablation, mice were randomly divided into 4 groups: Vehicle, ^131^I 2.775 MBq, ^131^I 5.55 MBq, and LID + ^131^I 2.775 MBq. The LID group was fed a LID for up to 7 days and then mice in the ^131^I 2.775, ^131^I 5.55, and LID + ^131^I 2.775 MBq groups were intravenously administrated with ^131^I, respectively. Scintigraphy imaging with ^99m^Tc pertechnetate was performed once in 2 weeks for 4 weeks. After establishment of athyroid mice, control or athyroid mice were injected with human anaplastic thyroid cancer cells co-expressing sodium iodine symporter and enhanced firefly luciferase (ARO/NF) to evaluate RAI uptake. Scintigraphy imaging with ^99m^Tc pertechnetate was performed with ARO/NF tumor-bearing mice. Scintigraphy imaging showed decreased thyroid uptake in the LID + ^131^I 2.775 MBq group compared to other groups. Scintigraphy images showed that tumor uptake was statically higher in athyroid mice than in control mice. These data suggest that these optimized conditions for thyroid ablation could be helpful to establish an *in vivo* mouse model.

## Introduction

Carcinoma of the thyroid gland is the most common endocrine malignancy (95%), representing approximately 1% of all malignancies in Western countries^1–3^. The frequency of thyroid carcinoma has been increasing worldwide over the past three decades^4,5^. For treatment of thyroid cancer, thyroidectomy is mainly used to remove cancerous lesions, and ^131^I may be administered to eradicate unresectable metastatic thyroid cancer^6–8^. Although ^131^I is an effective therapeutic modality for thyroid cancers that express sodium iodide symporter (NIS), a substantial percentage of metastatic thyroid carcinomas do not express adequate levels of the protein and are categorized as radioiodine refractory thyroid cancers. These cancers do not or respond poorly to chemotherapy and radiotherapy, and show poor prognosis compared to differentiated thyroid cancers^9–12^. Therefore, re-induction of NIS via pharmacologic interventions in radioiodine refractory thyroid cancer has been actively investigated to convert these cases into radioiodine sensitive thyroid cancers.

However, in order to optimize ^131^I therapy for treatment of radioiodine sensitive differentiated thyroid cancers and to discover NIS-inducing drugs for radioiodine refractory cancer, it is essential to develop an athyroid animal model of thyroid cancer. Murine models have gained popularity for a number of reasons, including small body size, rapid gestation period, relatively low maintenance costs, and an extensively characterized genome^13^. However, radioiodine treatment of thyroid cancer in normal mice results in the thyroid gland absorbing most of administered radioiodine, which may confound the experimental results.

Thyroid surgery is the most commonly used to develop athyroid mouse models. However, the surgery is a challenging technique owing to limited visualization and difficulties with precise manipulation of the surgical field; the surgical procedure may also damage nearby organs including the thymus and great vessels. Moreover, thyroid surgery remains limited in application and is practiced by a relatively small number of specialists^14,15^. Owing to its simplicity, safety, and cost-effectiveness, radioactive iodine (RAI; ^131^I) has been used clinically for more than 70 years to ablate the thyroid gland^16–18^. The success rate of the ablation is influenced by various factors, including total thyroid volume, body iodine pool, and thyroid-stimulating hormone (TSH) levels^19,20^. Even though it provides many benefits, reports of ^131^I thyroid ablation are rare for athyroid mouse models, and ^131^I thyroid ablation has not yet been optimized^21–23^. The reported ^131^I administration doses for thyroid ablation in mice is variable^21,24–26^. The body iodine pool is one of the important factors associated with successful radioiodine ablation, and low-iodine diets (LIDs) have been widely used to decrease the body iodine pool. The effect of LIDs on radioiodine ablation of patients with thyroid cancer has been investigated, but few studies have evaluated the effect of LIDs in athyroid models using radioiodine. The current study established an athyroid mouse model using ^131^I administration and also optimized the ^131^I thyroid ablation protocol in this mouse model.

## Results

### Influence of diet on thyroidal ^131^I uptake

**Figure 1:**
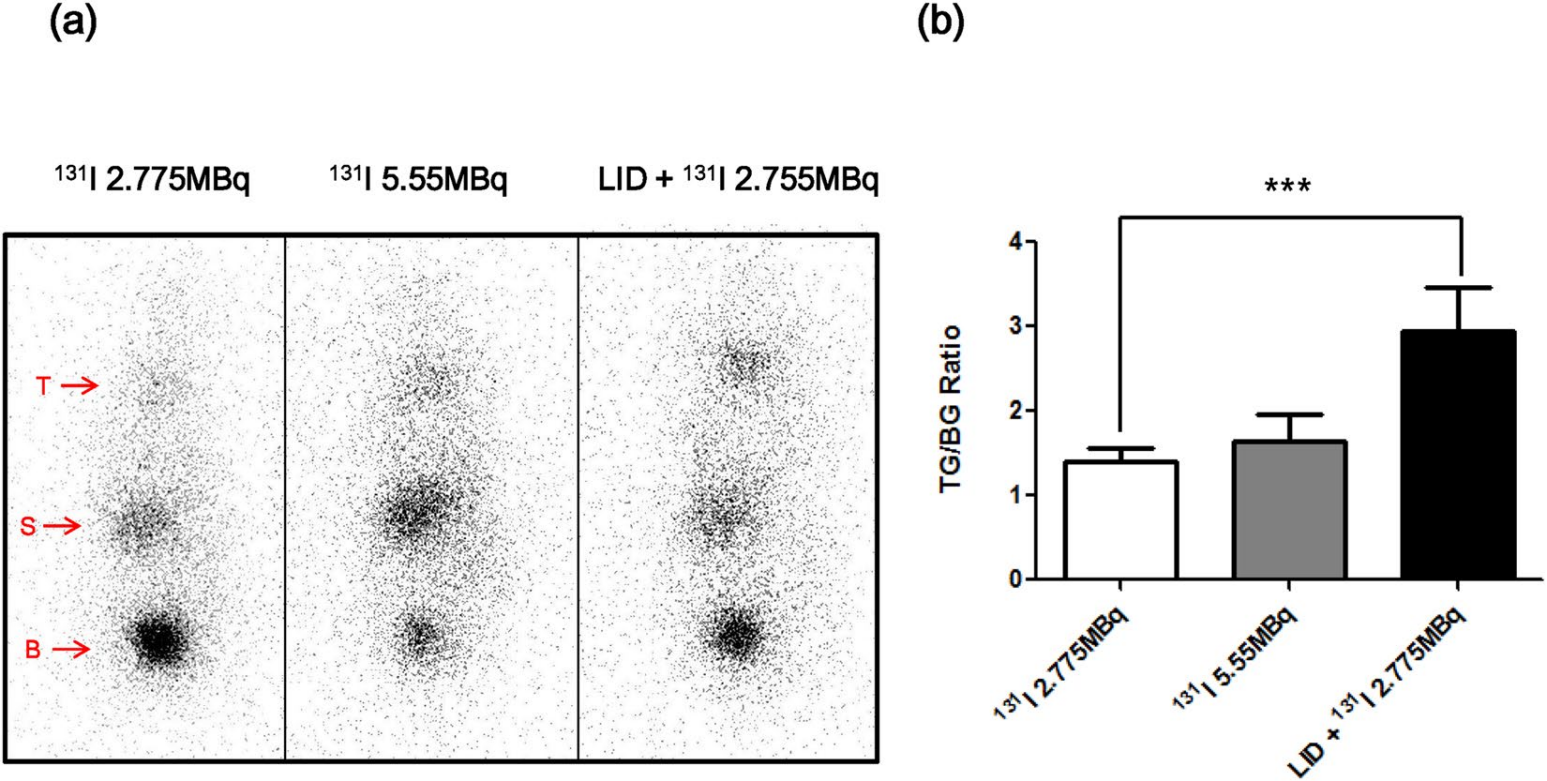
*In vivo* scintigraphy imaging after ^131^I treatment. (**a**) Gamma camera imaging was performed to determine ^131^I uptake in thyroid tissue. Physiological iodide uptake was shown in the thyroid (T), stomach (S), and urinary bladder (B). (**b**) Quantitation of radioiodine uptake in the thyroid. Data are expressed as % of thyroid gland (TG)/background (BG).

Before the experiment, the mice were divided into 4 treatment groups; (1) the vehicle group, (2) ^131^I 2.775 MBq, (3) ^131^I 5.55 MBq, (4) and LID + ^131^I 2.775 MBq. To evaluate the effect of LID effect in these mice, we monitored thyroid ^131^I uptake by gamma camera imaging. The LID + ^131^I 2.775 MBq group had considerably higher ^131^I accumulation than groups without LID, as shown in Fig. 1. There were no significant differences in body weight among the groups (Supplementary Fig. 2).

### ^99m^Tc pertechnetate scintigraphy imaging

**Figure 2:**
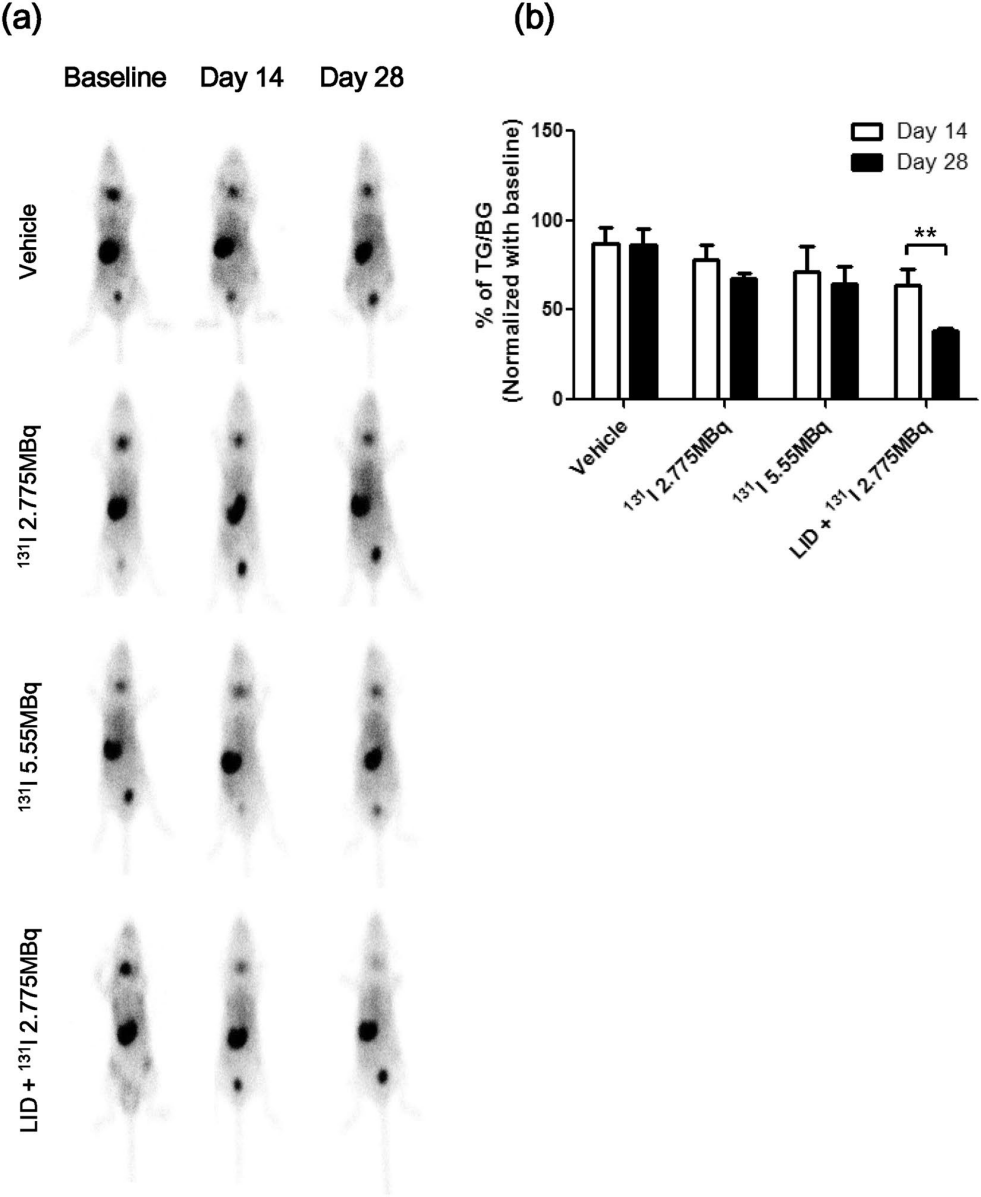
Scintigraphy imaging with ^99m^Tc pertechnetate to compare thyroid uptake. (**a**) *In vivo* gamma camera imaging was performed at baseline and days 14 and 28 following RAI treatment. (**b**) Quantitation of ^99m^Tc pertechnetate uptake in the thyroid. Data are expressed as % of thyroid gland (TG)/background (BG) after normalization using baseline values.

To evaluate the effects of thyroid ablation according to ^131^I treatment, we monitored gamma camera images of the thyroid gland using ^99m^Tc pertechnetate. There were no significant differences for 2 weeks after injection of ^131^I to ablate the thyroid. However, starting at 3 weeks, ^99m^Tc pertechnetate accumulation in the thyroid gland gradually decreased. As shown in Fig. 2, the LID + ^131^I 2.775 MBq group had greater reduction in thyroid ^99m^Tc pertechnetate accumulation compared to the RAI groups (^131^I 2.775 MBq, ^131^I 5.55 MBq, and LID + ^131^I 2.775 MBq: 68.0 ± 2.6, 64.6 ± 9.4 and 38.2 ± 1.6%TG/BG at day 28, respectively). Of note, ^99m^Tc pertechnetate accumulation in the LID + ^131^I 2.775 MBq group was significantly decreased when compared to the uptake in the RAI groups.

### Measurement of thyroid size using sonography

**Figure 3:**
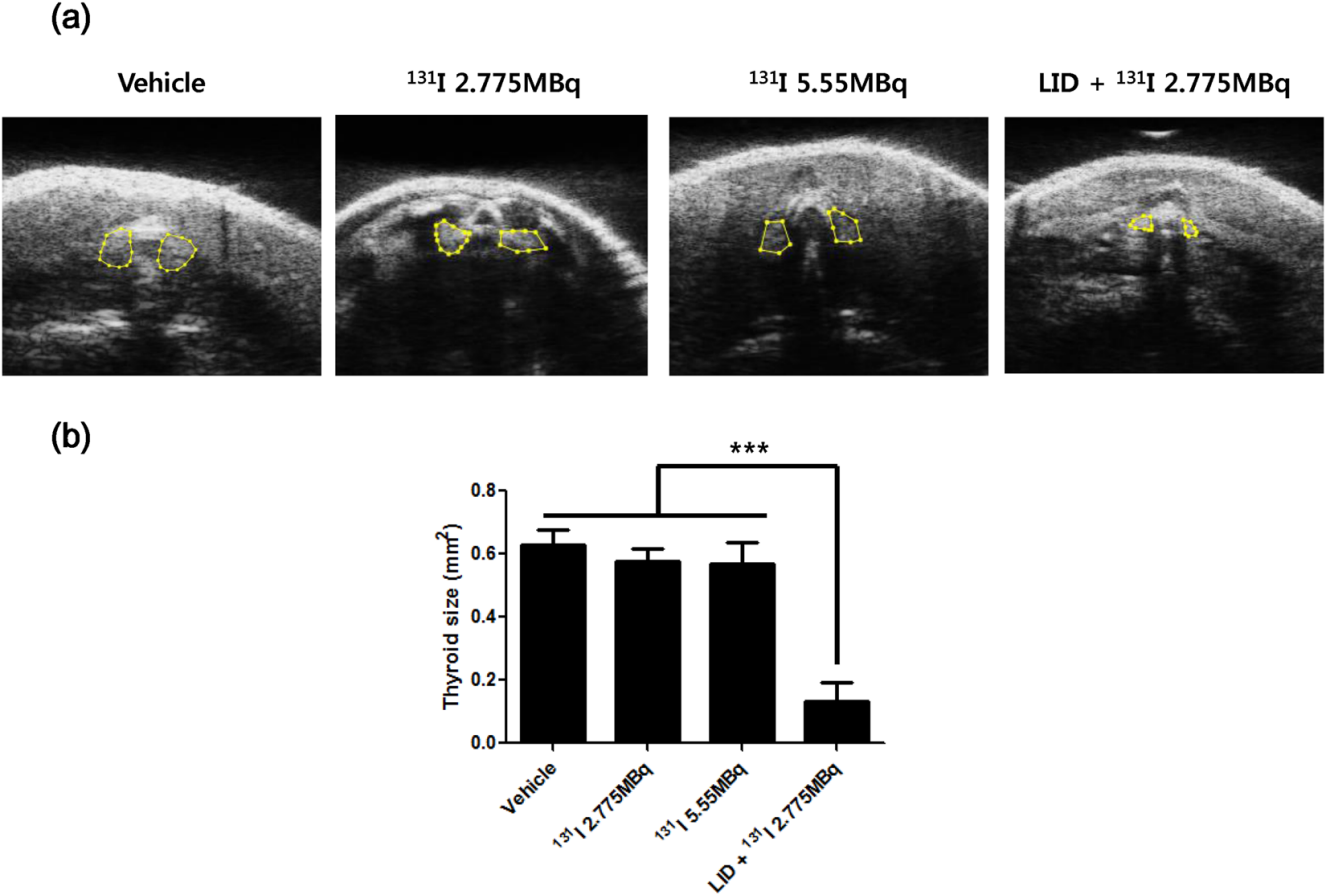
Measurement of thyroid size using sonography. (**a**) Thyroid size was assessed in all groups using small animal sonography with 40 MHz transducer. (**b**) Quantification data was shown in the bar graph. Values are means ± SD.

The sonographic results showed that thyroid size in LID + ^131^I 2.775 MBq group was significantly decreased compared to other groups (Thyroid areas; 0.13 ± 0.06 mm^2^, *p* value < 0.001) and there were no significant difference of thyroid size among vehicle,^131^I 2.775 and ^131^I 5.55 MBq groups (Vehicle vs ^131^I 2.775 MBq vs ^131^I 5.55 MBq; 0.63 ± 0.05 vs 0.58 ± 0.04 vs 0.57 ± 0.07 mm^2^, respectively, Fig. 3).

### Serum T4 hormone concentration

**Figure 4:**
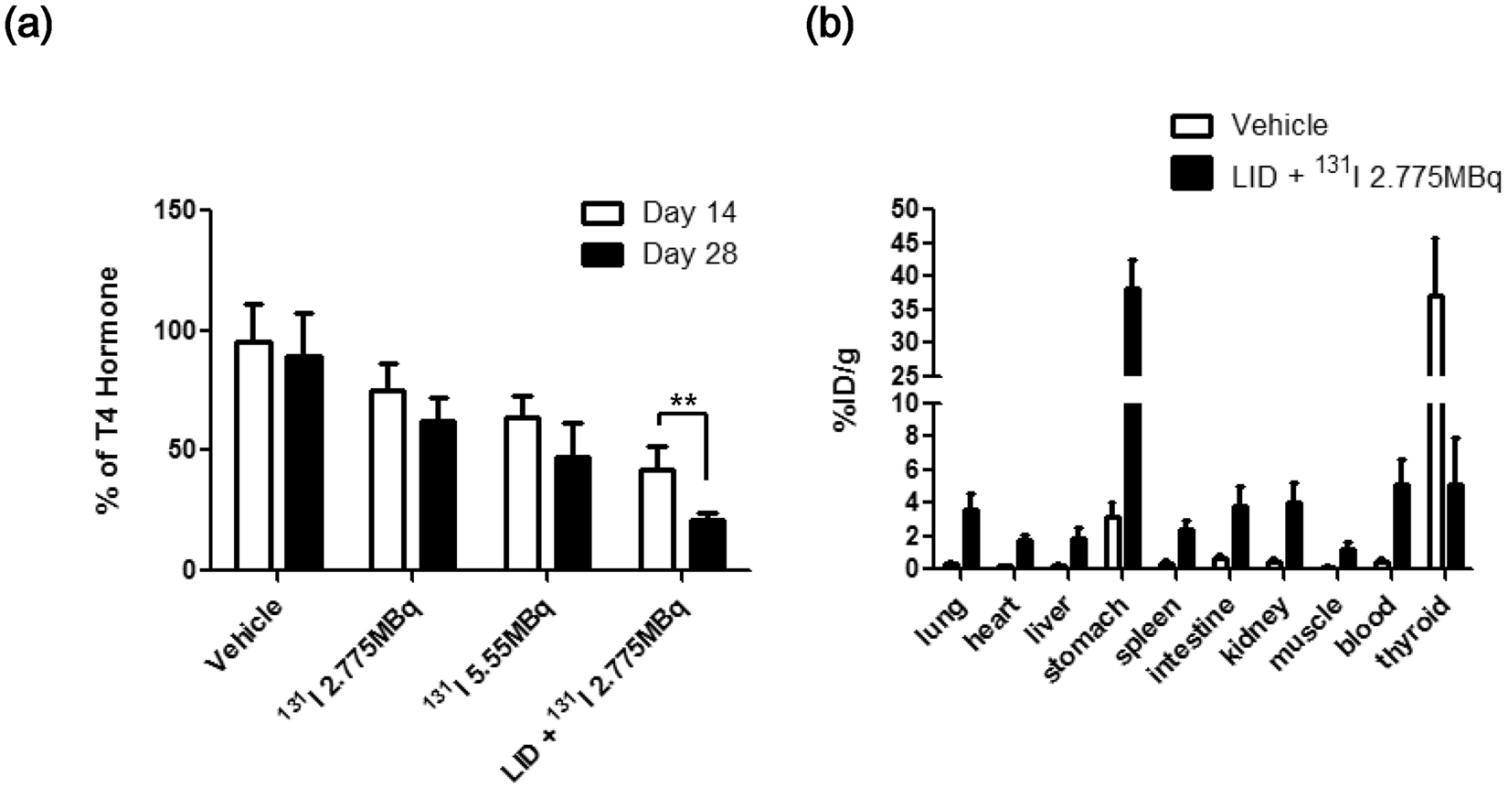
T4 hormone measurement and ^125^I bio-distribution. (**a**) On days 14 and 28, serum was collected from all groups. Serum T4 hormones were measured and normalized with those of the control group. Experiments were performed at least in triplicate; values are means ± SD. (**b**) Mice were intravenously injected with 1.85 MBq ^125^I. After 4 hours, the mice were sacrificed and radioactivity in dissected organs was measured. Data are expressed as percent injected dose per gram (%ID/g).

The concentration of serum T4 hormone constantly had declined over time in all groups except a vehicle group. ^131^I 2.775 MBq groups showed reduction of T4 hormone concentration. The serum T4 concentration of ^131^I 5.55 MBq group was lower than ^131^I 2.775 MBq groups and had dropped more than 50% at day 28. But there is no significantly difference of T4 hormone concentration in RAI groups between day 14 and 28. Interestingly, LID + ^131^I 2.775 MBq group revealed that T4 hormone concentration was dramatically decreased at day 14 and 28 compared to other groups (Day 14 vs 28; 42.4 ± 9.5 vs 20.8 ± 3.5% of T4 hormone, respectively, Fig. 4a).

### ^125^I bio-distribution study

Bio-distribution of^125^I was evaluated in order to validate thyroid uptake of radioactivity after establishment of the thyroid ablation model. As shown in Fig. 4b, thyroid uptake in the LID + ^131^I 2.775 MBq group was significantly lower compared with uptake in the vehicle group (vehicle, LID + ^131^I 2.775 MBq; 34.7 ± 7.8 vs. 5.11 ± 2.79% ID/g, respectively). Interestingly, uptake in other organs (lungs, heart, liver, stomach, etc.) was approximately 3–10-fold higher in the athyroid mice compared to the vehicle group.

### Histological analysis

**Figure 5:**
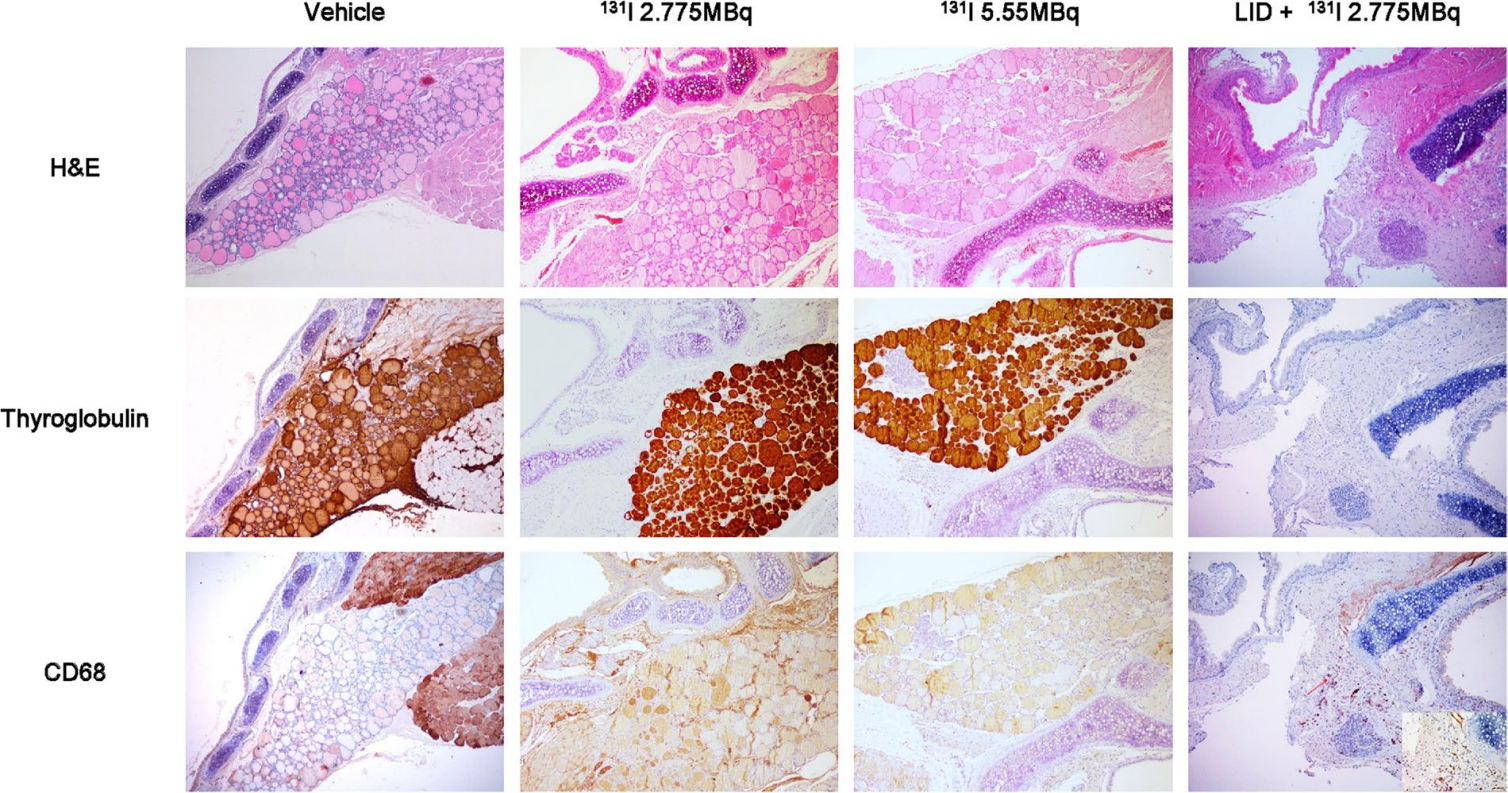
Immunohistochemical staining for thyroglobulin and CD68 proteins. On day 28, thyroid tissues were excised for immunohistochemical analysis and stained with thyroglobulin- or CD68-specific antibodies. In the LID + ^131^I 2.775 MBq group, no staining was observed in the thyroid gland on thyroglobulin immunostained slides (x100) and darkly stained CD68-positive macrophages (inlet, x400) were found more frequently in the ablated thyroid gland (arrow) compared with the normal thyroid gland of control group (x100).

H&E staining clearly showed thyroid follicles in vehicle, ^131^I 2.775 MBq and ^131^I 5.55 MBq groups, but not in the LID+ ^ 131^I 2.775 MBq group. In vehicle, ^131^I 2.775 MBq and ^131^I 5.55 MBq groups, immunohistochemical staining revealed strong staining with thyroglobulin in the follicular cells, but macrophages showing CD68 positive staining were sparse in the thyroid gland. On the other hand, ablated thyroid tissues in the LID+ ^ 131^I 2.775 MBq group did not show thyroglobulin staining. CD68 positive macrophages were found more frequently in the thyroid gland in the LID+ ^ 131^I 2.775 MBq group compared with the control group (Fig. 5).

### *In vivo*^99m^Tc pertechnetate imaging of NIS expressing tumor

**Figure 6:**
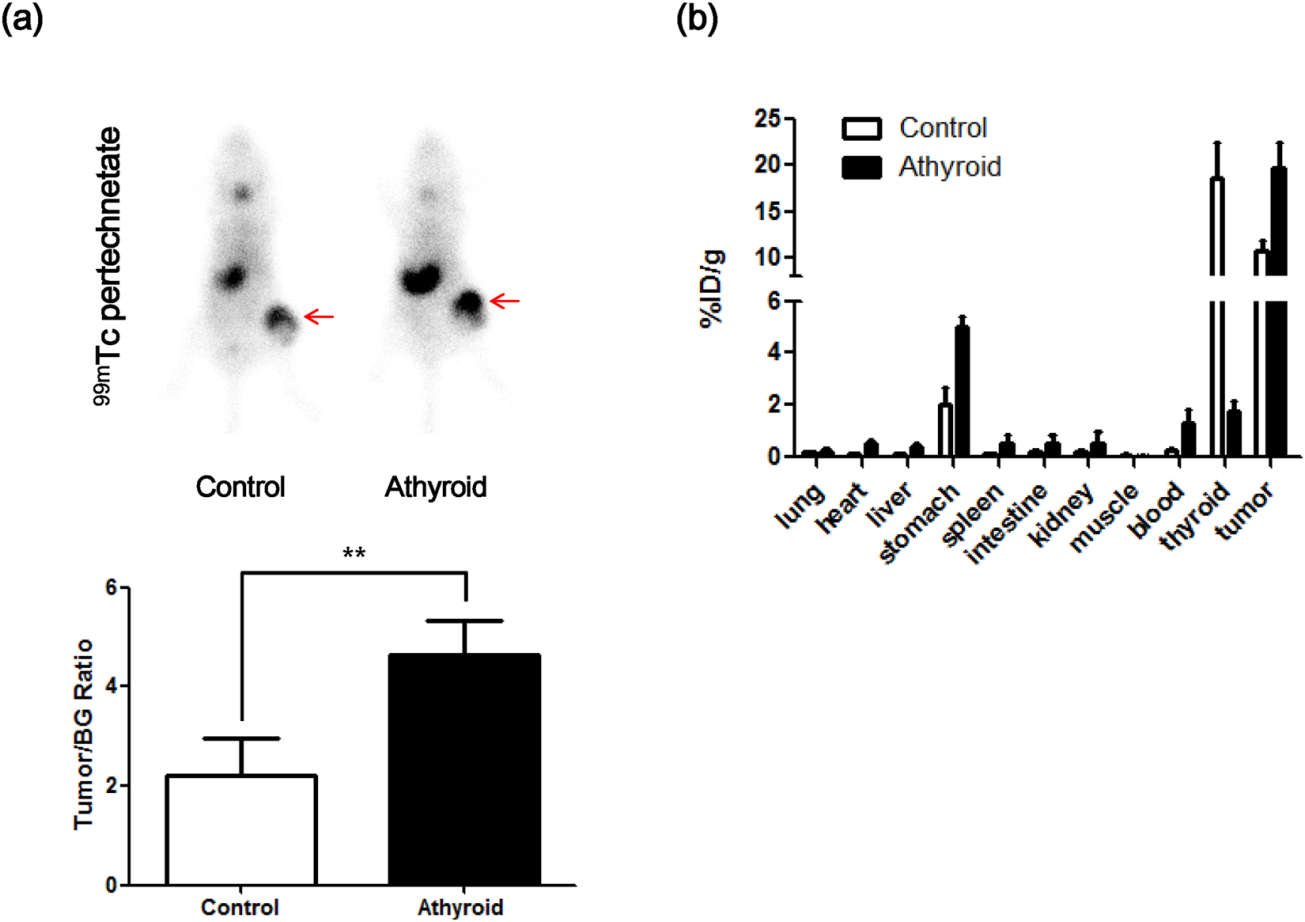
*In vivo* ^99m^Tc pertechnetate scintigraphy of tumor-bearing mice. (**a**) ARO/NF cells were inoculated in right hind thigh of mice after treatment with LID and RAI for 14 days. ^99m^Tc pertechnetate scintigraphy was performed in tumor-bearing mice (control and athyroid groups). Arrows indicate the location of ARO/NF tumor xenografts. (**b**) Quantitation of radioiodine uptake in the tumor area. Data are expressed as ratio of tumor (TG)/background (BG).

*In vivo* scintigraphy imaging with ^99m^Tc pertechnetate was performed to determine whether uptake of NIS-expressing tumors was affected by thyroid ablation. To normalize tumor uptake of ^99m^Tc pertechnetate, the bioluminescent signal of the tumor was measured after scintigraphy. The imaging revealed ^99m^Tc pertechnetate uptake in the thyroid gland, stomach, and urinary bladder, as well as NIS-expressing ARO/NF tumors. The control group showed lower tumor pertechnetate uptake compared to the athyroid group (2.20 ± 0.77 vs. 4.62 ± 0.91, respectively, *p* < 0.01, Fig. 6a).

### ^125^I bio-distribution study in NIS-expressing tumor mice

After ^99m^Tc pertechnetate uptake evaluation, ^125^I bio-distribution was assessed. Athyroid mice with NIS-expressing tumors exhibited increased ^125^I uptake in the tumor region compared with tumors in the control group (^125^I tumor uptake of control and athyroid groups: 10.6 ± 1.18 vs. 19.8 ± 2.67%ID/g, respectively, Fig. 6b). The athyroid mice group had lower accumulation of ^125^I in the thyroid region compared to the control group (^125^I thyroid uptake in the control and athyroid groups: 18.6 ± 3.81 vs. 1.76 ± 0.38, %ID/g, respectively).

## Discussion

Temporary LIDs are recommended before radioactive iodine treatment or scanning in thyroidectomized thyroid cancer patients. LIDs are widely used to deplete body iodine pools, which increases the availability of radioactive iodine to NIS-expressing tissues^27–30^. The American Thyroid Association (ATA) recommends an LID defined by an intake of <50 μg/day for 1–2 weeks before ^131^I administration, while the British Thyroid Association recommends an LID for 2 weeks before ^131^I administration^28,31^. However, there is no consensus about the necessity of LIDs for ablation of the entire thyroid gland, such as ^131^I treatment for Graves’ disease, and there are limited reports regarding the relationship between LID and ^131^I thyroid ablation outcomes in animal models.

In the current study, we developed an effective ^131^I thyroid ablation by performing an *in vivo* experiment to investigate the effects of an LID on radioiodine uptake in the thyroid gland. Prior to *in vivo* experiments, the determination of the minimum ^131^I dose for effective thyroid ablation is required. However,^131^I doses ranging from 1.04 to 37 MBq (28–1000 μCi) were used in previous thyroid ablation reports^21,24–26^. The ^131^I ablation dose can produce systemic and local adverse effects, including weight loss, salivary gland dysfunction, tracheal damage, and breathing difficulty^32,33^. Higher doses tends to show higher ablation success rates, but more frequent adverse effects^34^. Therefore, it is important to determine the optimal dose of ^131^I for successful thyroid ablation.

In order to optimize the dose of RAI for effective thyroid gland ablation, mice were divided into 4 groups: vehicle, ^131^I 2.775 MBq; ^131^I 5.55 MBq; and an LID + ^131^I 2.775 MBq. Scintigraphy imaging was performed with ^99m^Tc pertechnetate in all groups before ^131^I ablation to check the baseline status of the thyroid gland. Accumulation of ^99m^Tc pertechnetate or radioiodine was observed in tissues that endogenously express NIS, including the thyroid, stomach, and salivary glands, as well as the urinary bladder, which eliminates the radionuclide from the body. During administration of the LID, there were no significant differences in body weights between the groups. There were also no significant differences in body weight between the RAI (^131^I 2.775 and 5.55 MBq) and control groups. One week after administration of the LID, all mice were intravenously administrated ^131^I 2.775 or 5.55 MBq and gamma camera imaging was performed. Thyroid uptake of ^131^I on the gamma camera image was, at first, used as an indicator of successful intravenous injection of ^131^I and to quantitatively assess thyroid ^131^I accumulation. Accumulation of ^131^I in the thyroid gland was visualized in all groups, but more intense thyroid uptake was observed in the LID + RAI group compared to uptake in the other groups, which suggests that depleting the body iodine pool via an LID resulted in enhanced ^131^I accumulation in the thyroid gland, which increases ^131^I ablation by delivering high radiation doses to the thyroid gland.

In accordance with thyroid uptake of ^131^I, scintigraphy imaging with ^99m^Tc pertechnetate 4 weeks after ^131^I ablation revealed significantly lower thyroid uptake of ^99m^Tc pertechnetate in the LID + RAI group compared with uptake in the non-LID groups and we further measured thyroid size and serum T4 levels to confirm the thyroid ablation effect, these results revealed decreased thyroid size and T4 level in LID + RAI group compared with other groups. In addition, we investigated the biodistribution of ^125^I and histological changes in the thyroid region. The results of the bio-distribution assessment using ^125^I showed that the LID + RAI group had lower radioactivity in the ablated thyroid gland and higher radioactivity in other organs, including the lungs, heart, liver, and stomach, compared to levels in the vehicle group. Immunohistochemical analysis using thyroglobulin staining confirmed more prominent destruction of thyroid follicles in the LID + RAI group. In contrast, we observed increased density of CD68-positive macrophages in the thyroid gland. We assumed that the dead follicular cells and cellular debris resulting from the radioiodine ablation are phagocytosed by macrophages. The degree of CD68 expression in damaged thyroid tissue might be related to the degree of damage in thyroid follicular cells^35^.

The results of ^99m^Tc pertechnetate scans for NIS-expressing tumors revealed significantly increased uptake in the tumors and other organs in athyroid mice compared to control mice. These scans revealed almost faint uptake in the thyroid bed of the athyroid mice, indicative of successful ^131^I thyroid ablation; the thyroid ablation also significantly increased tracer uptake in the NIS-expressing tumor xenograft. The biodistribution of radioiodine in the athyroid mouse model is more similar to that observed in thyroidectomized thyroid cancer patients than euthyroid mouse models; therefore, the athyroid mouse model might be more appropriate for developing radioiodine therapy than other euthyroid mouse models.

Overall, we successfully optimized conditions for thyroid ablation with a week of LID and low dose of RAI. Our results could be useful for investigation of RAI refractory thyroid cancers in mouse models that mimic real clinical situations by removing the thyroid gland before ^131^I therapy.

## Materials and Methods

### Animals

Female Balb/c nude mice, 5.5 weeks old and weighing averages of 18.7 ± 0.46 g (mean ± SD) were purchased (Hamamatsu, Shizuoka, Japan). The mice were maintained under specific pathogen-free conditions in order to adapt to the experimental conditions for 1 week before starting the experiment. The animals were maintained at room temperature (20–25 °C) at 40–70% relative humidity.

### Experimental protocol

All procedures were reviewed and approved by the Kyungpook National University Animal Care and Use Committee, and performed in accordance with the Guiding Principles for the Care and Use of Laboratory Animals. Mice were randomly divided into different treatment groups for thyroid ablation: (1) the vehicle group (n = 10), which received no ^131^I and a LID, (2) ^131^I treatment group (n = 10), which received ^131^I 2.775 MBq by intravenous injection, (3) ^131^I treatment group (n = 10), which received ^131^I 5.55 MBq by intravenous injection, (4) ^131^I treatment combined with LID group (n = 10), which received a LID for 7 days prior to radioiodine administration as well as ^131^I 2.775 MBq by intravenous injection. The body weights of the mice were measured once a week before gamma imaging. Blood samples were taken to estimate T4 concentration at days 0, 14, and 28. Gamma camera images of the thyroid were obtained following intravenous injection of ^99m^Tc pertechnetate (18.5–22.2 MBq) at days −1, 14, and 28 after ^131^I administration, respectively. At the end of the experiments, the animals were sacrificed and ^125^I bio-distribution was performed. The tissues were analyzed by hematoxylin and eosin (H&E) staining and immunohistochemistry. A schematic of thyroid ablation procedure is shown in Supplementary Fig. 1.

### ^99m^Tc pertechnetate whole-body imaging

To evaluate the efficacy of the thyroid ablation, whole-body scans were performed with ^99m^Tc-pertechnetate. All mice were administrated with ^99m^Tc-pertechnetate (18.5–22.2 MBq) and static gamma camera images were obtained for 20 min using a 2 mm pinhole collimator (Infinia II, GE Healthcare, Milwaukee, WI, USA). Additionally, ^131^I gamma camera images were obtained at that time of ^131^I injection to confirm thyroid uptake of ^131^I. The mice were maintained under isoflurane (Forane, ChoongWae Co., Seoul, Korea) anesthesia during injection and scanning. We set regions of interest (ROIs) in the thyroid region of the mice to obtain a total count of thyroid uptake. Background activity was measured using circular ROI of the same size on the head. Quantification of thyroid uptake was defined as the % of thyroid gland (TG)/background (BG), which was calculated as the total count of thyroid uptake divided by the total background count.

### Sonography of the thyroid gland

Ultrasonography of the thyroid gland was performed to measure size of the gland non-invasively using Prospect 3.0 ultrasound imaging system (S-Sharp Corporation, New Taipei, Taiwan). Mice were anesthetized with 1–2% isoflurane in 100% O_2_ and then placed in ventral side up position. B mode imaging with 40 MHz transducer was acquired on thyroid area of mice after replenishment of the ultrasound gel on the neck of mice.

### Serum T4 measurement

Serum T4 measurements were made using serum samples pooled from retro orbital blood puncture, as described previously^36^. On days −1, 14, and 28, serum T4 concentrations were estimated by radioimmunoassay using RIA-gnost-T4 Kits (Cisbio Bioassays, France) according to the manufacturer’s instructions. Serum samples and standard solutions with radiolabelled tracer were incubated in antibody-coated test tube. After incubating for 2 h, the solution was removed. A gamma counter (Cobra II, Packard, Perkin Elmer, MA, USA) was used to measure the radioactivity adhering to the tube.

### Histologic examination

The mice were sacrificed by cervical vertebrae dislocation on day 29 after ^131^I administration. After the anterior neck was incised vertically, the thyroid lobes were excised and fixed with 4% formalin overnight. The specimens were embedded in paraffin, sectioned into 4-µm-thick sections and mounted on slides. The specimen section slides were deparaffinized and stained using H&E. Immunohistochemical staining for thyroglobulin (Abcam, Cambridge, MA, USA) at a 1:250 dilution and CD68 (Abcam) at a 1:200 dilution were performed. The H&E and immunohistochemical stained slides were analyzed by light microscopy.

### Bio-distribution of ^125^I

The mice were intravenously injected with 1.85 MBq of ^125^I. Four hours later, blood samples were taken from all mice and the mice were sacrificed. *Ex vivo* radioactivity measurements were taken from the lungs, heart, liver, stomach, spleen, intestine, kidney, muscle, and thyroid using a gamma-counter (Cobra II). The data were expressed as the percentage injected dose per gram tissue (%ID/g).

### Statistical analysis

All data are expressed as means ± standard deviation (SD) and were statistically analyzed by *t*-test using GraphPad Prism 5, version 5.01 (GraphPad Software, Inc. USA). *P* values less than 0.05 were considered statistically significant.

## Electronic supplementary material

Supplementary information
