# Supplementary material for: Development of an athyroid mouse model using 131I ablation after preparation with a low-iodine diet
Source: Sci Rep. 2017 Oct 16;7:13284. doi: 10.1038/s41598-017-13772-8 (PMC5643325; doi:10.1038/s41598-017-13772-8)

Supplementary info

Development of an athyroid mouse model using ^{131}I ablation after preparation with a low-iodine diet

Ji Min Oh[1] , Ho Won Lee[1], Senthilkumar Kalimuthu[1], Prakash Gangadaran[1], Se Hwan Baek[1], Man- Hoon Han[2], Chae Moon Hong[1], Shin Young Jeong[1], Sang-Woo Lee[1], Jaetae Lee[1, 3] and Byeong-Cheol Ahn[1 *]

[1]Department of Nuclear Medicine, Kyungpook National University and Hospital, Daegu, South Korea

[2]Department of Pathology, Kyungpook National University and Hospital, Daegu, South Korea

[3]Daegu-Gyeongbuk Medical Innovation Foundation (DGMIF), 80 Chembok-ro, Dong-gu, Daegu, South Korea

Correspondence

Byeong-Cheol Ahn, M.D., Ph.D.
Department of Nuclear Medicine, School of medicine, Kyungpook National University Hospital
50 Samduk 2-ga, Daegu 700-721
Republic of Korea
E-mail: abc2000@knu.ac.kr

Supplementary Materials and Methods

Establishment of anaplastic thyroid cancer cell line expressing NIS

A human anaplastic thyroid cancer cell line (ARO cells) was maintained in RPMI medium (Hyclone, Logan, UT, USA) containing 10% fetal bovine serum (Hyclone) and 1% penicillin–streptomycin (Gibco, Carlsbad, CA, USA) at 37°C in a 5% CO_2 humidified atmosphere. ARO cells were transfected with a recombinant lentivirus containing both human sodium iodine symporter (NIS) and green fluorescent protein (GFP). GFP-positive cells were sorted by flow cytometry (FACSorter, BD Biosciences, CA, USA). The stable cells expressing NIS and GFP were transduced with retrovirus having both enhanced firefly luciferase (effluc) and Thy1.1. Thy1.1-positive cells were enriched with magnetic beads (Miltenyi Biotec, Bergisch Gladbach, Germany). The line of cells with stable expression of NIS, GFP, effluc, and thy1.1 is referred as ARO/NF cells.

Luciferase assay

To perform the luciferase assay, parental ARO and ARO/NF cells were plated in 96-well white plates and cultured with growth medium. After 24 hours, 3 µL of D-luciferin (30 mg/mL) was added to each well and the luciferase activity was measured using a microplate luminometer (Molecular Devices, Sunnyvale, CA, USA).

Radioiodide uptake assay

For iodine uptake, parental ARO and ARO/NF cells were plated in 24-well plates. The ^{125}I uptake level was determined by incubating the cells with 500 µL of Hank's balanced

salt solution (HBSS) containing 0.5% bovine serum albumin (bHBSS), 3.7 kBq carrier-free I-125 and 10 μmol/L sodium iodide (specific activity of 740 MBq/mmol) at 37°C for 30 minutes. After incubation, the cells were washed twice as quickly as possible with ice-cold bHBSS buffer and detached using 500 μL of trypsin. The radioactivity was measured using a gamma-counter (Cobra II, Packard, Perkin Elmer, MA, USA).

Cell proliferation assay

Cell proliferation was determined using a Cell Counting Kit (CCK)-8 (Dojindo Laboratories, Tokyo, Japan). To examine cell proliferation, the parental ARO cells and ARO/NF cells were plated at 2×10^4 per well in 96-well plates. Two days later, a 20 μL cell count solution was added to each well and the plates were incubated at 37ºC for 1 hour. The absorbance was measured at 450 nm using a microplate reader. (Bio-Rad Laboratories, Hercules, CA).

Tumor transplantation

To determine the ^{99m}Tc pertechnetate uptake of NIS-expressing tumors, control and athyroid mice were randomly selected and challenged with ARO/NF cells (5×10^6) in right flank of mice for 14 days. After 14 days, gamma camera images were obtained with ^{99m}Tc pertechnetate (18.5–22.2 MBq). After gamma camera imaging, bioluminescence imaging (BLI) with IVIS Lumina III (Perkin-Elmer, Wellesley, MA, USA) was performed to measure the tumor size by effluc signal.

Therapeutic effect of RAI on NIS-expressing tumor

To evaluate the therapeutic effect of RAI on ARO/NF tumor bearing mice, ^{131}I 22.2MBq was administrated when tumor volume reached around 100mm^3 and then scintigraphic imaging was acquired. In addition, BLI was performed at day 0, 5 and 10 after the administration of ^{131}I

Supplementary Results

Establishment of NIS and effluc expressing ARO cells

Flow cytomeric analysis showed that the stably transfected cells highly expressed the NIS and effluc genes. ^{125}I uptake by ARO/NF cells increased according to cell number, whereas ^{125}I uptake by parental ARO cells remained at the basal level. Radioiodine uptake in ARO/NF cells was 23.6 times higher than in parental ARO cells. A luciferase assay of the ARO and ARO/NF cells was performed. The bioluminescence signal was about 446 times higher in ARO/NF than parental ARO cells. The results of the cell proliferation assay showed that there was no significant difference in cell proliferation activity between parental ARO and ARO/NF cells

Enhanced therapeutic effect of RAI on NIS-expressing tumor in athyroid mouse model

Scintigraphy with ^{131}I showed that tumoral uptake of ^{131}I was much higher in athyroid group than in control group. BLI was used to determine the therapeutic effects of ^{131}I on ARO/NF tumors. BLI was performed at day 0, 5 and 10 after treatment of ^{131}I. , Athyroid group showed lower BLI signal from the tumor compared to control group

Supplementary Figure legends

Supplementary Fig. 1 Experimental schematic of the thyroid ablation procedure

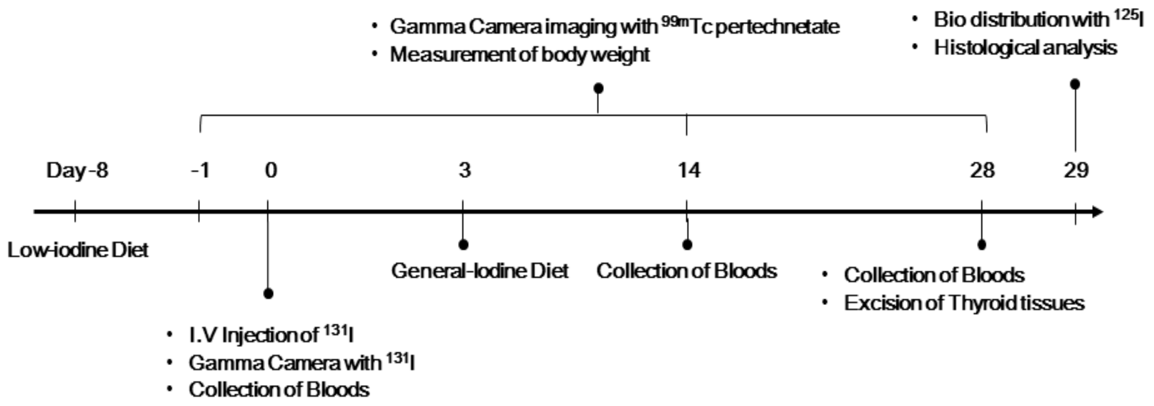

Supplementary Fig. 2 Measurement of body weight

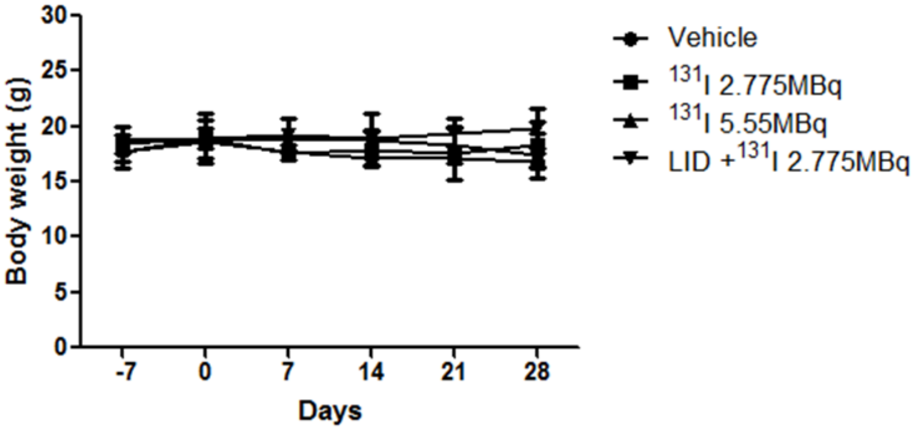

Supplementary Fig. 3 Establishment of cancer cell line that stably express both NIS and effluc genes.

a) *In vitro* ^{125}I uptake in ARO and ARO/NF cells. ^{125}I uptake was measured after incubation with bHBSS containing 3.7 kBq carrier-free I-125 and 10 µM NaI for 30 minutes. b) *In vitro* luciferase assay with ARO and ARO/NF cells using a microplate luminometer. c) Cell proliferation rates in ARO and ARO/NF cells. Cell proliferation was determined using a CCK assay two days after cell seeding. Data are expressed as means ± SD.

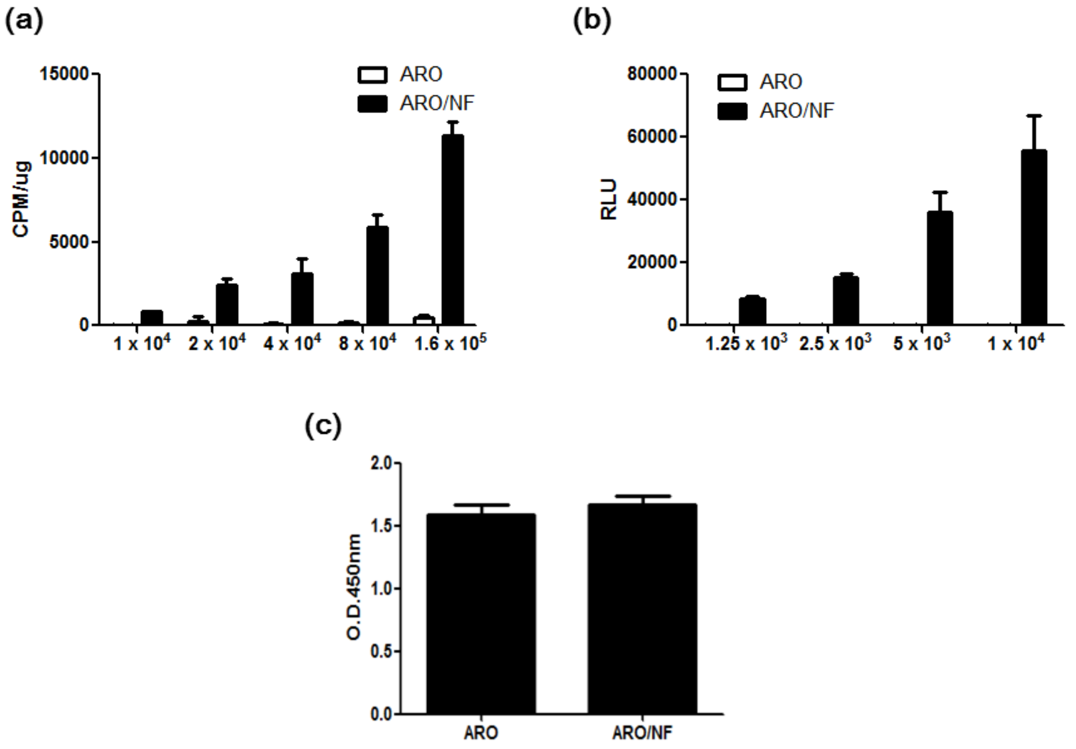

Supplementary Fig. 4 *In vivo* bioluminescence imaging of ARO/NF tumor-bearing mice

a) Control and athyroid mice were administrated ARO/NF cells in right thigh, and *in vivo* bioluminescence imaging performed. b) Quantification of tumor bioluminescence signals. Data are expressed as means ± SD.

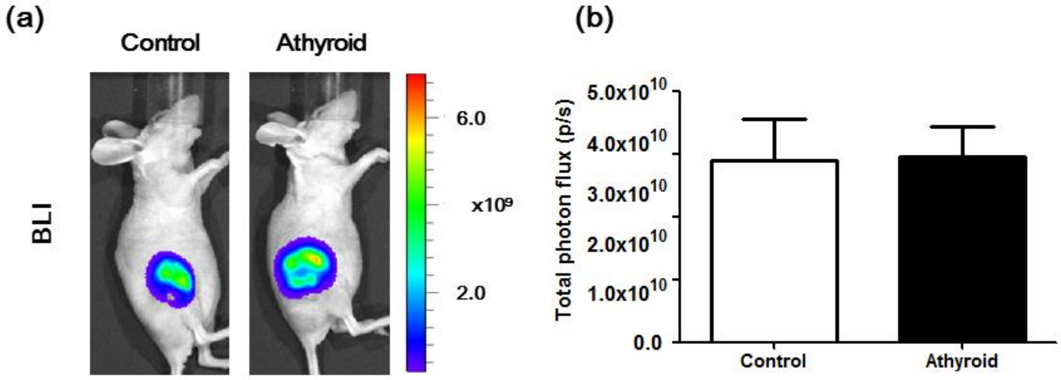

Supplementary Fig. 5 *In vivo* monitoring of RAI therapeutic effect on NIS-expressing tumor xenograft

a) Mice were administrated with ^{131}I 22.2MBq and then scintigraphy was conducted. Athyroid mouse demonstrated higher radioactive iodine in the tumor xenograft compared to control mouse. b) BLI was performed until day 10 post treatment of ^{131}I 22.2MBq. Therapeutic effect of the ^{131}I was higher in Athyroid mouse compared to control mouse, due to higher tumoral uptake of ^{131}I in the Athyroid mouse.

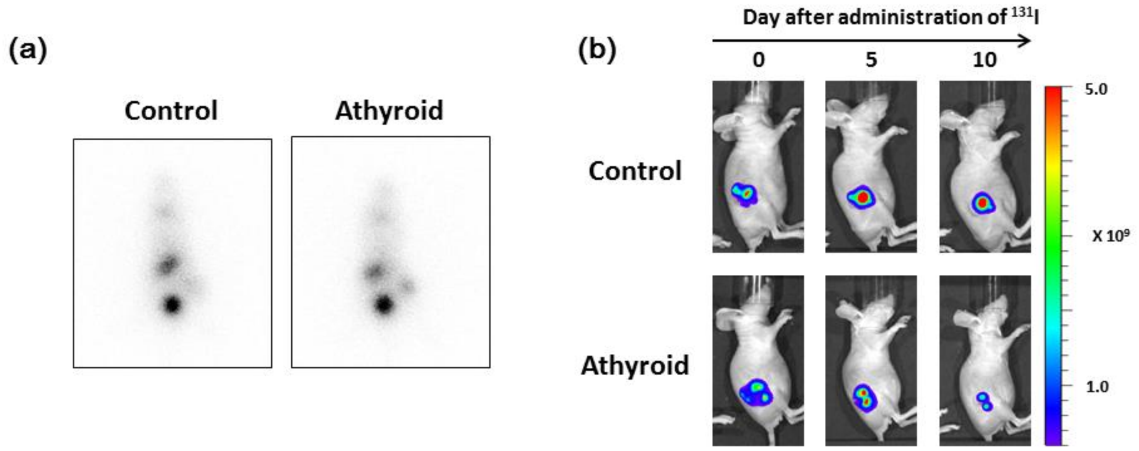

Supplement: Supplementary file 1 — Supplementary information [file 41598_2017_13772_MOESM1_ESM.pdf]
